# Multiaction Antimicrobial, Anti-inflammatory, and Prohealing Hydrogel as a Novel Strategy for Preventing Postoperative Pancreatic Fistula

**DOI:** 10.34133/bmr.0194

**Published:** 2025-04-23

**Authors:** Yuan Zhou, Lan Li, Fangsheng Chen, Tingting Huang, Maoen Pan, Heguang Huang

**Affiliations:** ^1^ Department of General Surgery, Fujian Medical University Union Hospital, Fuzhou 350001, China.; ^2^ Key Laboratory of Optoelectronic Materials Chemical and Physics, Fujian Institute of Research on the Structure of Matter, Chinese Academy of Sciences, Fuzhou 350002, China.

## Abstract

Postoperative pancreatic fistula remains a challenging complication after pancreaticoduodenectomy. Addressing this issue requires effective strategies to promote anastomotic healing. In this study, we developed a novel hydrogel designed to close pancreaticoenteric anastomosis after pancreaticoduodenectomy. The hydrogel—composed of polyvinyl alcohol, chitosan, and dopamine-modified oxidized hyaluronic acid—exhibited excellent antibacterial, anti-inflammatory, and wound healing properties. It was designed to conform well to the anastomotic site for clinical application. The hydrogel demonstrated good biocompatibility, appropriate mechanical strength, low swelling, and strong adhesive properties, meeting specific requirements for pancreaticoenteric anastomosis environments. Moreover, by activating the cell cycle, it promoted cell proliferation and migration, thereby accelerating anastomotic closure. Addition of the potent broad-spectrum antibiotic meropenem further enhanced its antibacterial efficacy, targeting common microbial species involved in delayed healing and fistula formation after pancreatic surgery. In a rat model of pancreatic fistula, the hydrogel effectively sealed the anastomosis, filled potential suture gaps, and exerted antibacterial, anti-inflammatory, and tissue regeneration–promoting effects around the anastomotic site. Therefore, this hydrogel, with its ideal degradation properties, shows promising application prospects in closing pancreaticoenteric anastomosis following pancreaticoduodenectomy, thereby offering an effective solution to reduce complications such as pancreatic fistula after pancreatic surgery.

## Introduction

Pancreaticoduodenectomy (PD) remains the standard surgical approach for treating benign and malignant tumors of the pancreatic head and cancers of the periampullary region [1]. However, PD carries a high complication rate of 30% to 50%, with postoperative pancreatic fistula (POPF) being a serious concern, leading to mortality in 0% to 5% of cases [2]. Despite advances in surgical techniques and perioperative management, it is still challenging to effectively reduce the incidence of POPF [3]. The pancreaticoenteric anastomosis is a high-risk area prone to rupture and leakage due to factors such as high digestive enzyme activity, potential microbial contamination, and tissue mechanical stress, markedly affecting postoperative recovery and survival [4].

In response to these challenges, clinicians have explored various techniques such as modified anastomotic methods (e.g., duct-to-mucosa, “U”-shaped suture, fish-mouth anastomosis, and Blumgart technique) to mitigate POPF, but with varying success rates [5]. Recent studies have investigated the use of biomaterials, such as stents, fibrin sealants, and mesh implants, to reinforce pancreaticoenteric anastomosis; however, these approaches have shown limited efficacy [6–8]. Hydrogels, emerging as promising biomaterials, have garnered attention for their excellent biocompatibility, mechanical properties, and drug delivery capabilities, gradually finding application in pancreatic-related diseases [9].

Hyaluronic acid (HA), known for its biocompatibility and bioactivity in wound healing and tissue regeneration, lacks sufficient tissue adhesion and mechanical strength for direct intraoperative use [10,11]. Inspired by mussel adhesion, dopamine-modified oxidized hyaluronic acid (OHAD) enhances adhesive properties through dopamine grafting, effectively adhering to tissue surfaces and forming a protective barrier against microbial invasion [12]. This is achieved by promoting focal adhesion, a crucial mechanism for the initial attachment of cells, including fibroblasts and endothelial cells, to the hydrogel surface. This facilitates the establishment of stable tissue integration and encourages cellular migration, both of which are key for anastomotic healing and the prevention of POPF [13,14]. However, single OHAD’s mechanical strength and biological performance may be insufficient for clinical requirements. To address this issue, a hydrogel system combining polyvinyl alcohol (PVA) and chitosan (CS) was developed. PVA was selected for its mechanical stability and freeze–thaw crosslinking capability, while CS as a polysaccharide derived from shellfish offers antibacterial properties and promotes wound healing, aiding in anastomotic healing and reducing inflammation [15,16]. In particular, it prevents microbial invasion at the pancreaticoenteric anastomosis site, which is a key factor in preventing delayed wound healing and POPF. Furthermore, CS aids in promoting wound healing by encouraging cell migration and tissue regeneration. This is achieved by its inherent ability to enhance cell adhesion to the hydrogel surface, ensuring stable anastomosis closure [17].

Research by Kimura et al.[18] has highlighted bacterial contamination around the pancreas as a trigger for POPF, with *Enterococcus*, *Pseudomonas aeruginosa*, and *Escherichia coli* as common pathogens [19,20]. Meropenem (MP) was selected as a loading agent primarily because of the broad-spectrum antibiotic that provides local antimicrobial activity when loaded into a hydrogel, and there is evidence that its antimicrobial spectrum covers both the causative and resistant flora of postoperative infections and reduces the incidence of POPF, particularly in cases of B/C grade [18,21].

**Fig. 1.**
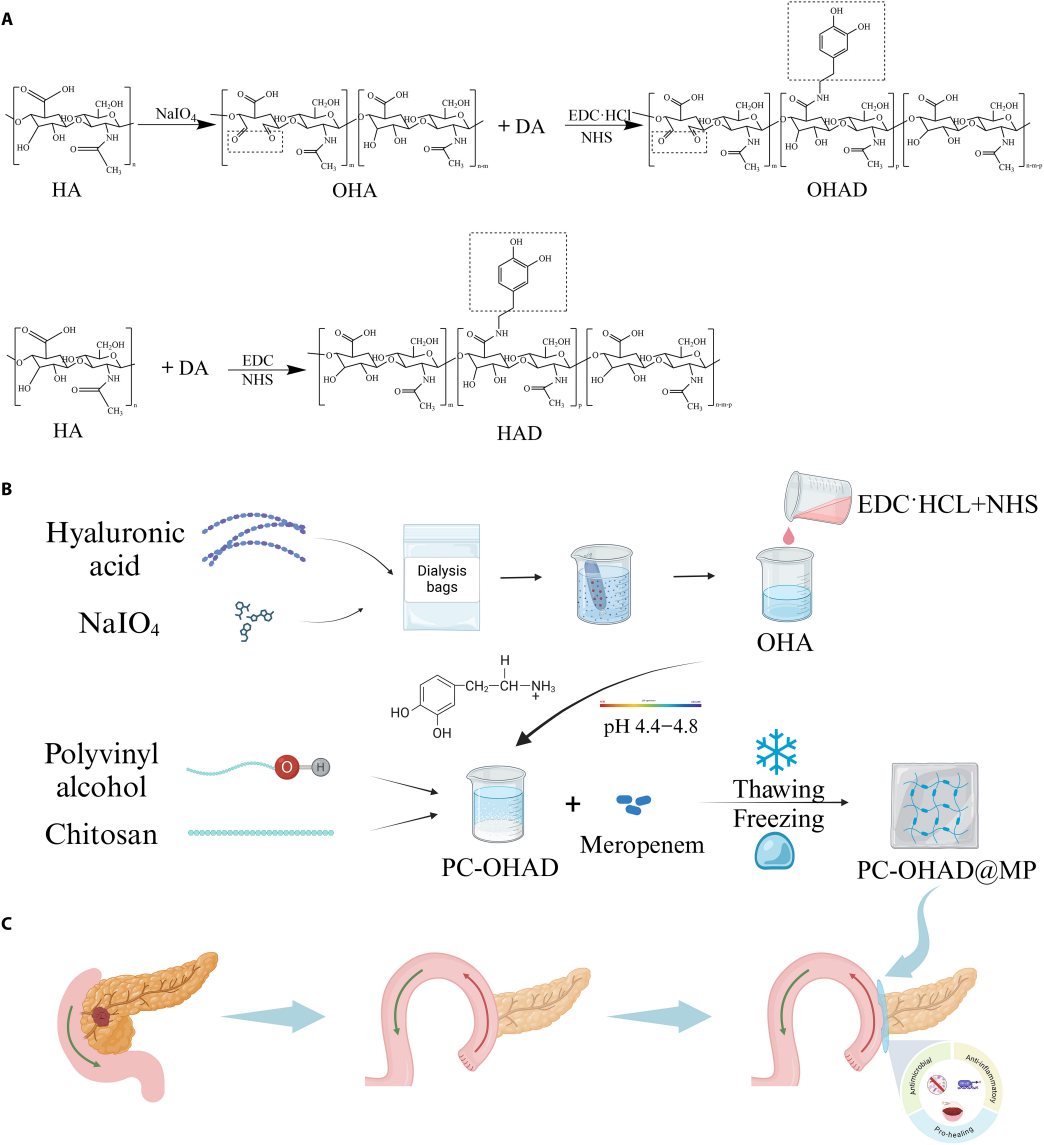
Preparation process and applications of hydrogels. (A) Modification and modification process of hyaluronic acid. (B) Schematic diagram of the hydrogel preparation process. (C) Main application scenarios and methods of hydrogels.

Unlike previous approaches relying on single-component PVA or polyglycolic acid (PGA) hydrogels for mechanical reinforcement, our design integrates dopamine-modified OHAD to enhance tissue adhesion and CS for synergistic antimicrobial effects. This multifunctional strategy addresses the limitations of existing materials, which often lack combined mechanical stability, antibacterial activity, and prohealing properties [22]. As shown in Fig. 1C, we hypothesized that this novel PVA/CS/OHAD hydrogel system would not only seal anastomotic gaps but also actively modulate the wound microenvironment to reduce POPF incidence. The significance of this study, which innovatively developed a multifunctional hydrogel that combines antimicrobial, anti-inflammatory, and mechanical reinforcement properties, is to provide a comprehensive solution to the key challenges of preventing POPF and improving postoperative recovery from pancreatic surgery.

## Materials and Methods

### Materials

PVA (PVA 1799, average molecular weight [M_W_] ≈ 77 kDa, ≥98%), CS (M_W_ ≈ 130 kDa, deacetylation degree ≥85%), and HA (M_W_ ≈ ~400 kDa) were purchased from Aladdin (Shanghai, China). TUNEL (terminal deoxynucleotidyl transferase–mediated deoxyuridine triphosphate nick end labeling) assay kit, live-dead double staining kit, annexin V-APC/7-AAD apoptosis kit, and cell cycle flow cytometry kit were obtained from Elabscience Biotechnology Co., Ltd. (Wuhan, China). *Staphylococcus aureus*, *E. coli*, *P. aeruginosa*, *Klebsiella pneumoniae*, mouse fibroblast cells (L929), and mouse leukemia monocytic macrophage cells (RAW264.7) were provided by the American Type Culture Collection (ATCC, Maryland, USA). Antibodies against p65, p-p65, IκBα, p-IκBα, IKKβ, Cyclin A2, Cyclin D1, Cyclin E2, Cdk2, c-Myc, GAPDH, α-tubulin, and β-actin were provided by Abcam, Ltd. (Cambridge, UK), Cell Signaling Technology, Inc. (Massachusetts, USA), and ProteinTech Wuhan Sanying Biotechnology (Wuhan, China).

The simulated pancreatic fluid was configured as in previous studies [23], and a solution containing 0.1% trypsin and 0.5% sodium chloride was prepared in deionized water, and the pH was adjusted to 8.0 by adding NaOH.

### Synthesis of hydrogel

#### Synthesis of oxidized hyaluronic acid

A total of 1.0 g of HA was dissolved in 100 ml of ultrapure water to achieve a concentration of 10 mg/ml. Sodium periodate (0.535 g, 2.5 mM) was added, and the reaction mixture was stirred in the dark at 25 °C for 6 h. Ethylene glycol (140 μl, 2.5 mM) was added to quench any unreacted periodate, terminating the oxidation reaction. The reaction mixture was then stirred further in the dark at room temperature for 1 h. Subsequently, the mixture was transferred into a dialysis bag (molecular weight cut off [MWCO], 3.5 kDa) and dialyzed thoroughly against ultrapure water for 4 days, with water changes every 4 to 6 h to remove low-molecular-weight impurities. Finally, the dialyzed solution was freeze-dried for 3 days to obtain oxidized hyaluronic acid (OHA).

#### Synthesis of OHAD

A total of 1 mmol OHA was dissolved in distilled water and stirred at room temperature until a viscous, transparent OHA solution was obtained. To this solution, 2 mmol EDC·HCl and 1 mmol NHS were added. The pH of the reaction mixture was adjusted to 4.4 to 4.8 using 0.1 M NaOH or HCl solution, followed by stirring at room temperature for 30 min to fully activate the carboxyl groups of HA. Subsequently, dopamine hydrochloride (1.00 mmol, 153.2 mg) was added to the reaction mixture, and the mixture was stirred overnight under nitrogen. After completion of the reaction, unreacted dopamine hydrochloride was removed by dialysis against 0.01 M HCl aqueous solution (MWCO, 3.5 kDa) for 2 days, followed by dialysis against distilled water for 1 day. Finally, the solution was freeze-dried for 3 days to obtain a sponge-like white solid of OHAD [14] (Synthesis of oxidized hyaluronic acid and OHAD see Fig. 1A).

#### Synthesis of PVA/CS/OHAD hydrogel loaded with MP

First, OHAD was added to phosphate-buffered saline (PBS) solution at pH 7.4 to obtain OHAD solutions of 3, 6, and 9 wt%. Concurrently, CS was added to a 2 wt% glycolic acid and stirred for 12 h to obtain a 1 wt% CS solution. Simultaneously, PVA was dispersed and dissolved in deionized water at 95 °C for 4 h under vigorous stirring to obtain a 10 wt% PVA solution, and cooled to room temperature. The OHAD, CS, and PVA solutions were then mixed to achieve a mass ratio of OHAD/CS/PVA as 1:1:2. The mixture was stirred uniformly for 2 h, followed by centrifugation at 5,000 rpm for 5 min to remove bubbles. Subsequently, 0.17 mg/ml MP was added to the mixed solution to successfully prepare the PVA/CS/OHAD hydrogel loaded with MP (PC-OHAD@MP). Finally, the mixed solution was cast into designed molds and subjected to 3 freeze–thaw cycles (frozen at −20 °C for 20 h, thawed at 25 °C for 4 h) to obtain hydrogel, named PC-OHAD@MP (see specific process in Fig. 1B).

### Characterization of hydrogel

The structures of PVA, CS, HA, OHA, HAD, OHAD, and the synthesized hydrogel were characterized using Fourier-transform infrared spectroscopy (FTIR) with a Lambda950 instrument from Perkin Elmer (USA). The oxidation degree of HA and OHA, and the grafting rate of dopamine in HAD and OHAD were quantitatively determined using proton nuclear magnetic resonance (^1^H NMR) on an ECZ400S instrument (Japan). Scanning electron microscopy (SEM) images of the hydrogel surface morphology were obtained using SU-8010 equipment (Hitachi, Japan).

### Swelling behavior

Swelling analysis aimed to determine the maximum swelling ratio of hydrogels. The swelling studies were conducted at 37 °C. The initial weight of the developed hydrogel was designated as *W*_0_. Subsequently, equal-weight hydrogel samples were placed into 2 ml of PBS. Every 4 h, the hydrogel was removed, excess surface water was blotted dry with tissue paper, and the swollen weight (*W*_S_) of the hydrogel was measured. Every hour, 1 ml of fresh PBS was added to the hydrogel sample until the swollen weight of the hydrogel no longer increased. The swelling weight percentage (%SW) of the hydrogel formulation was calculated using Eq. 1. Three replicates of every group were carried out to obtain the mean value.

$$\%\mathrm{SW}=\left(W_{S}-W_{0}\right)/W_{0}\times 100\%$$ (1)

### In vitro degradation study

Considering the typical pH range of 5 to 9 in wound environments and the optimal pH of approximately 8.0 for enzymes like pancreatin, the degradation behavior of the hydrogel was evaluated at pH values of 5, 7.4, 8, and simulated pancreatic fluid. Hydrogel samples of identical cylindrical shape (diameter 11.6 mm, height 2.5 mm) from different components were freeze-dried, and their initial weights (*W*_0_) were measured. These samples were immersed in 20 ml of buffer solutions with different pH values at 37 °C, rotated at 100 rpm. At regular intervals, the hydrogels were removed from the buffer solution, dried with filter paper, freeze-dried again, and weighed (*W*_t_). Additionally, considering that local temperatures may increase due to inflammatory reactions at wound sites [24], we further tested the degradation of hydrogels at different temperatures, namely 25, 37, and 42 °C. The remaining weight percentage (%) of the hydrogel was calculated using Eq. 2. Three replicates of every group were carried out to obtain the mean value.

$$\text{Remaining weight percentage}\;\left(\%\right)=W_{t}/W_{0}\times 100\%$$ (2)

### Tensile study

To measure the mechanical properties of the hydrogel, single-axis tensile tests were conducted at 25 °C using a universal materials testing machine (AG-X plus, SHIMADZU). The hydrogel was cast into dumbbell-shaped samples using polytetrafluoroethylene molds. During the experiment, the samples were subjected to a constant tensile speed of 30 mm/min to measure tensile strength and fracture strain. At least 5 samples of each hydrogel type were tested to ensure reliability and consistency of results.

### Cumulative release of MP

To incorporate the antibiotic into the hydrogel matrix, the direct loading method was employed. Hydrogels were prepared using a known concentration of MP solution (0.17 mg/ml), and their release profiles were evaluated. Dried drug-loaded samples were placed into 1.5 ml of pH 5.4 PBS solution, pH 7.4 PBS solution, and simulated pancreatic fluid at 37 °C to study the in vitro release of the antibiotic. The released amount of the antibiotic was measured every 24 h using spectrophotometry at a wavelength of 220 nm with a SpectraMax i3X multimode microplate reader (Molecular Devices). The experiment was conducted over 8 days, and all measurements were performed in triplicate for accuracy and reliability.

### In vitro cytocompatibility evaluation of the hydrogels

We centrifuged 1 ml of rat blood at 3,000 rpm for 10 min, discarded the upper serum layer, and washed the samples 3 times with PBS. We incubated 20 μl of red blood cells with different formulations of hydrogels at 37 °C for 4 h, using 0.1% Triton X-100 and PBS as positive and negative controls, respectively. The absorbance of different solutions at 540 nm was calculated using Eq. 3, where *A*_t_, *A*_pc_, and *A*_nc_ represent the absorbance at 540 nm for the test sample, positive control, and negative control, respectively.

$$\text{Hemolysis}\;\left(\%\right)=\left(A_{t}-A_{\mathrm{nc}}\right)/\left(A_{\mathrm{pc}}-A_{\mathrm{nc}}\right)\times 100\%$$ (3)

We evaluated the cytotoxicity of the hydrogels on L929 fibroblast cells using the CCK-8 method. Different hydrogel formulations were soaked in complete culture medium overnight to obtain extracts. L929 cells (5,000 cells/well) were inoculated into a 96-well plate and cultured overnight. The culture medium was replaced with extracts from different hydrogel formulations, and complete culture medium was used as a control. At predetermined time points, we removed the culture medium, added CCK-8 reagent, and incubated for 2 h. We measured the absorbance at 450 nm using a multifunction microplate reader (Molecular Devices, SpectraMax i3X) and calculated cell viability (%) using Eq. 4.

$$\text{Cell viability}\;\left(\%\right)=\left({\mathrm{OD}}_{\mathrm{sample}}-{\mathrm{OD}}_{\mathrm{blank}}\right)/\left({\mathrm{OD}}_{\mathrm{control}}-{\mathrm{OD}}_{\mathrm{blank}}\right)\times 100\%$$ (4)

The cytotoxicity of different hydrogel formulations on L929 fibroblast cells was assessed using the live/dead cell double staining method. L929 cells (1.5 × 10^5^ cells/well) were inoculated into 6-well plates and cultured in complete culture medium (MEM supplemented with 10% FBS) for 24 h and complete medium was replaced with hydrogel extracts, with each well supplemented with 2 ml of culture medium. We used L929 cells cultured in complete culture medium as controls. Following the manufacturer’s instructions, we applied live/dead cell staining kit and observed the cells under an inverted fluorescence microscope (Leica, Leica DMi8). We repeated each test 3 times for accuracy.

L929 cells (1.5 × 10^5^ cells/well) were seeded into 6-well plates precoated with cell climbing slices. After 24 h of incubation, apoptosis assay was performed according to the TUNEL assay kit. Images were acquired using an inverted fluorescence microscope (Leica DMi8). All experimental samples were in triplicate.

### Flow cytometry

L929 cells were seeded in a 12-well plate and cocultured with the extracted solutions from different components of hydrogels for 24 h. Following the manufacturer’s instructions, we applied the cell cycle flow cytometry kit and the annexin V-APC/7-AAD apoptosis kit for cell cycle analysis and apoptosis detection (BD Accuri C6 Plus). All experimental samples were in triplicate.

### Cell migration

Initially, L929 cells were seeded into a 6-well plate. After 24 h of incubation, a straight line was created in the center of each well using a 10-μl pipette tip, followed by washing twice with PBS. The cells were then treated with the extraction solutions from different component hydrogels, with L929 cells cultured in complete medium serving as controls. Images were captured under an inverted microscope (Leica DMi1) at 0, 6, 12, 24, and 48 h after treatment. All experimental samples were in triplicate.

### Antibacterial study

The antimicrobial activity of different component hydrogels against *S. aureus*, *E. coli*, *P. aeruginosa*, and *K. pneumoniae* was evaluated using the agar well diffusion method. Initially, 50 μl of bacterial suspension containing 1×10^8^ CFU/ml was spread onto LB agar plates. Subsequently, circular hydrogel discs with a diameter of 6 mm and a thickness of 1 mm, freshly prepared and of fixed dimensions, were placed onto the LB agar plates. After incubation at 37 °C for 24 h, the diameter of the inhibition zones was measured. Three replicates of every group were carried out to obtain the mean value.

### In vivo pancreatic fistula models and treatment

All animal experiments were strictly conducted following the National Research Council’s Guide for the Care and Use of Laboratory Animals and were approved by the Experimental Animal Ethics Committee of Fujian Medical University (IACUC FJMU 2024-Y-1849). Thirty-six-week-old male Sprague–Dawley rats (clean grade, weighing approximately 200 g) were randomly assigned to 3 groups: control (*n* = 10), Neoveil (*n* = 10), and PC-OHAD@MP (*n* = 10). Each group was further randomly divided into 3-day and 7-day subgroups, with 5 rats per subgroup. The rats were anesthetized using isoflurane. Prior to surgery, body weights were measured and recorded. The rats were placed supine, and the surgical model was adjusted based on previous methods by exposing and preserving the splenic artery and vein, and severing the pancreatic duct and surrounding parenchyma to induce pancreatic fistulae [25]. After pancreatic and ductal resection, Neoveil and PC-OHAD@MP were applied around the surgical site and secured in place with 4-0 absorbable sutures. The control group was directly sutured using 4-0 absorbable sutures [26]. The pancreatic tissue was repositioned after surgery, and the abdominal cavity was closed in layers. The rats were observed during recovery and housed under standardized conditions postoperatively. On postoperative days 3 and 7, rat body weights were remeasured, and photographs were taken at the suture site after re-entering the abdominal cavity under general anesthesia. Blood samples were collected to measure hematological parameters, C-reactive protein (CRP), alanine aminotransferase (ALT), aspartate aminotransferase (AST), urea, creatinine (CREA), and amylase levels. Peritoneal lavage was performed with saline to collect ascitic fluid for measuring ascitic amylase levels. Finally, tissue samples were collected from the pancreatic stump and various organs (heart, liver, spleen, lungs, and kidneys) for histological staining.

### Statistical analysis

Statistical analyses were performed using independent-samples *t* tests for 2 groups and one-way analysis of variance (ANOVA) for multiple groups. Experiments were repeated at least 3 times to obtain data, which are presented as mean ± SD. A *P* value lower than 0.05 was considered significant and denoted using asterisks as follows: ****P < 0.0001, ***P < 0.001, **P < 0.01, *P < 0.05.

## Results and Discussion

### Preparation and characterization of hydrogels

**Fig. 2.**
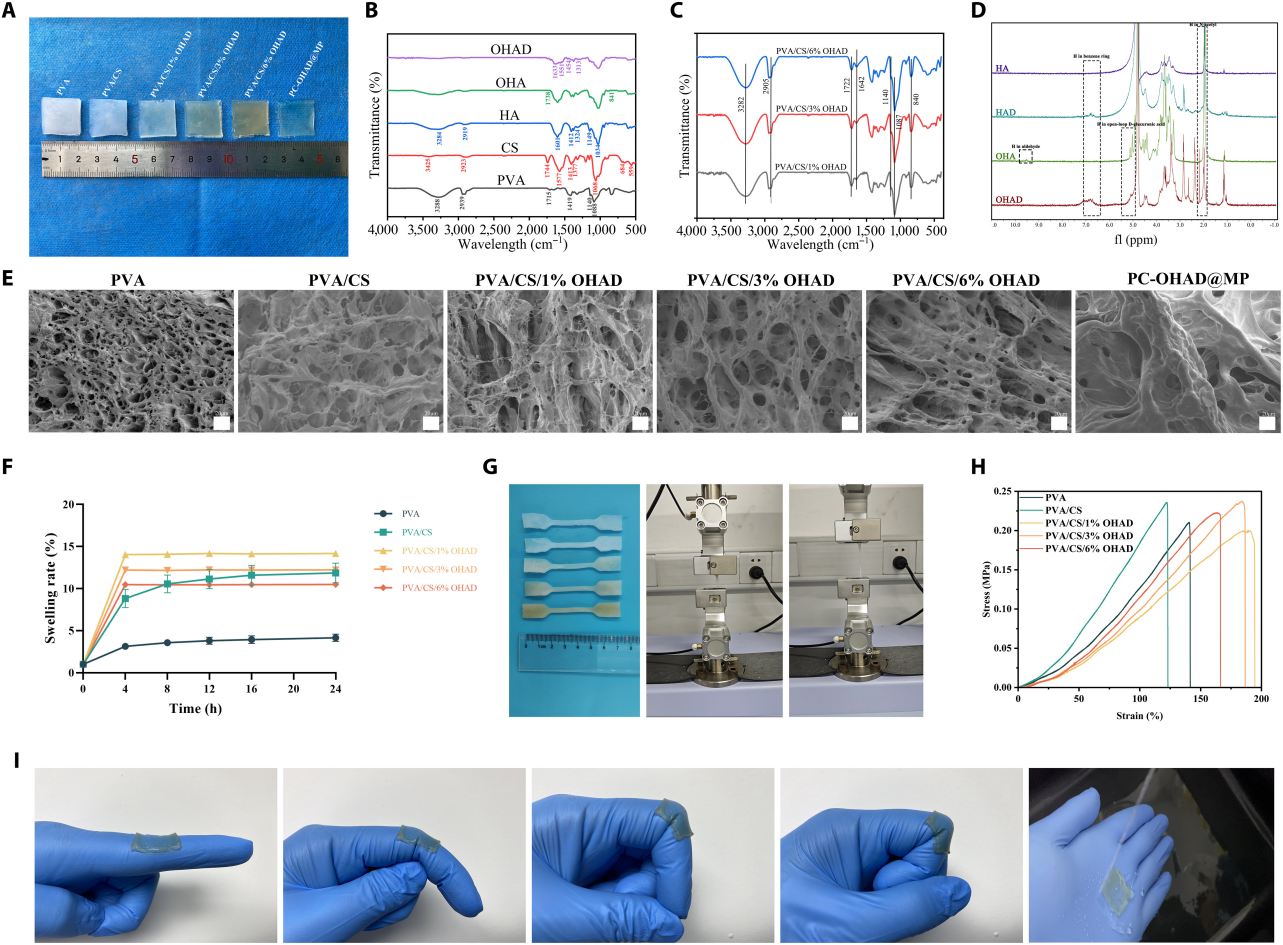
Appearance and characterization of hydrogels. (A) Appearance of the hydrogel (digital camera view). (B) FTIR spectra of hydrogels with single different components. (C) FTIR spectra of synthesized hydrogels with different OHAD concentrations. (D) ^1^H NMR spectra of HA, HAD, OHA, and OHAD. (E) Electron microscopy images of hydrogels with different compositions. (F) Swelling curves of hydrogels with different compositions (*n* = 3). (G) Preparation of tensile samples of hydrogels with different compositions and experimental process (*n* = 5). (H) Stress–strain curves of hydrogel samples with different compositions. (I) Adhesive properties of hydrogels in different scenarios.

As shown in Fig. 2A, the PVA hydrogel appeared white and opaque. The PVA/CS hydrogel was lighter and slightly more transparent compared with pure PVA, while the PVA/CS/OHAD hydrogel became progressively more transparent and changed from white to yellow with increasing OHAD concentration, culminating in the PC-OHAD hydrogel, which appeared slightly yellow and highly transparent.

In Fig. 2B and C, the FTIR spectrum of the PVA/CS/OHAD hydrogel reveals several key peaks: the 3,282 cm^−1^ peak corresponds to the stretching vibration of hydroxyl groups (–OH), from PVA or CS; the 2,905 cm^−1^ peak corresponds to methyl (–CH3) and methylene (–CH2–) groups, likely from PVA or dopamine [27]; the 1,722 cm^−1^ peak indicates carbonyl (–C=O) stretching, suggesting ester bonds or oxidation; the 1,642 cm^−1^ peak corresponds to carboxyl (–COO–) groups from HA or CS [28]; the 1,140 cm^−1^ and 1,087 cm^−1^ peaks are from C–O–C and C–C stretching, reflecting contributions from the sugar ring structures in CS; and the 840 cm^−1^ peak represents the bending vibration of sugar rings from CS. These peaks confirm the chemical structure of the PVA/CS/OHAD hydrogel [14,29].

Further confirmation of HA modification was achieved through ^1^H NMR spectroscopy (Fig. 2D), showing peaks at 9.48 parts per million (ppm; aldehyde protons) and 4.88, 4.99, and 5.09 ppm (protons of open-ring D-glucuronic acid), confirming successful OHAD synthesis. Peaks at 6.65, 6.73, and 6.78 ppm were attributed to aromatic protons of dopamine. A peak at 1.92 ppm corresponds to aldehyde or N-acetyl group protons [30]. Therefore, based on appearance, FTIR, and ^1^H NMR results, successful preparation of PVA/CS/OHAD and its various components was confirmed.

Figure 2E depicts the internal morphology of the developed hydrogels, illustrating highly porous structures with interconnected 3-dimensional networks. These interconnected pores help maintain the shape of the hydrogel and facilitate the transport of nutrients and metabolites within the hydrogel network, as well as absorption of exudate at the anastomosis site. PVA hydrogels exhibited small and dense pores, correlating with their poor water absorption, low viscosity, and strong mechanical properties. Addition of CS increased pore size, allowing for greater absorption of exudate around the anastomosis site [27]. Introduction of OHAD led to a uniform distribution of pore sizes, with SEM images of PC-OHAD@MP showing most pores occupied by MP.

Swelling behavior of different hydrogel compositions was evaluated in deionized water at 25 °C (Fig. 2F). PVA/CS/OHAD hydrogels reached swelling equilibrium within 4 h, with PVA and PVA/CS reaching equilibrium after 12 h. After 24 h, swelling percentages were as follows: PVA (4.2% ± 0.4%), PVA/CS (11.9% ± 1.2%), PVA/CS/1% OHAD (14.2% ± 0.4%), PVA/CS/3% OHAD (12.2% ± 0.2%), and PVA/CS/6% OHAD (10.5% ± 0.3%). Increasing OHAD concentration led to a gradual decrease in swelling percentage, which is beneficial for in vivo applications, particularly at intestinal anastomosis sites, where excessive swelling can compress surrounding tissues and affect normal physiological function, leading to complications [31].

Similarly, ideal anastomotic dressings must possess certain mechanical properties to avoid tissue damage and infection due to poor fitting at the anastomotic site. Figure 2G illustrates tensile samples prepared from PVA, PVA/CS, PVA/CS/1% OHAD, PVA/CS/3% OHAD, and PVA/CS/6% OHAD, with corresponding stress–strain curves shown in Fig. 2H. Addition of OHAD improved mechanical properties of the hydrogels compared with PVA and PVA/CS, albeit with a gradual decrease in tensile strength as OHAD content increased. PVA/CS/1% OHAD exhibited a tensile strength of 0.2 MPa and an elongation of 194%. PVA/CS/3% OHAD showed a tensile strength of 0.24 MPa and an elongation of 186%. PVA/CS/6% OHAD had a tensile strength of 0.19 MPa and elongation of 199%. To ensure optimal adherence at the anastomotic site, hydrogels must possess adequate adhesive properties. PC-OHAD@MP hydrogels adhered tightly to fingers with different bending angles (0°, 45°, 90°, and 135°) and remained stable when exposed to flowing water (Fig. 2I). Adhesion is critical for the clinical application of pancreatic closure materials, and although the introduction of dopamine can provide some adhesion to hydrogel systems, to prioritize biocompatibility and avoid potential toxicity from chemical crosslinkers (e.g., glutaraldehyde), our hydrogel was synthesized via physical freeze–thaw cycles, which inherently limits adhesion strength. For this reason, one of the future research directions is to further optimize its synthesis method and try to improve the adhesion properties in order to improve the structure and composition of hydrogels and enhance their adhesion. Additionally, the hydrogel exhibited resilience, rapidly returning to its original shape after compression (Fig. S1). These results demonstrate that the hydrogel possesses low swelling, good mechanical properties, and adhesive capabilities, meeting the requirements for intestinal anastomosis healing.

### Degradation and MP release from hydrogels

**Fig. 3.**
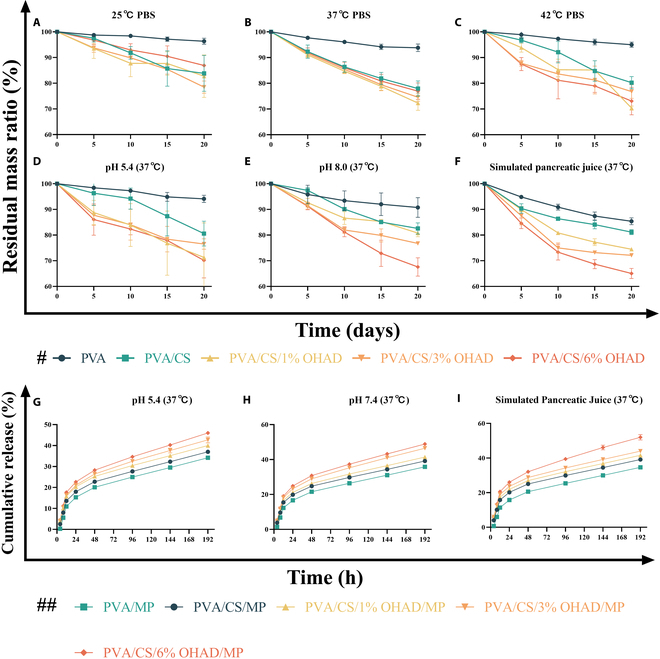
Degradation and loaded drug release profiles of hydrogels under different conditions (# indicates panels A to F; ## indicates panels G to I) (*n* = 3).

Considering that healing intestinal anastomosis usually ranges from 3 to 6 months, it is desirable that the degradation time of the hydrogel we developed corresponds to that time [6]. This process is crucial for the stability of dressings, as they continuously interact with surrounding tissues within specified intervals. As depicted in Fig. 3A to C, we found that the degradation rate of PVA was significantly lower than that of PVA/CS, PVA/CS/1% OHAD, PVA/CS/3% OHAD, and PVA/CS/6% OHAD at different temperatures, with the addition of OHAD enhancing the degradation rate. This observation aligns with findings by Oustadi et al. [32], who noted increased degradation with decreasing PVA concentration due to the lack of crosslinking and entrapment within the hydrogel structure, leading to fragmentation of PVA chains. Similarly, degradation experiments were conducted under different pH conditions (Fig. 3D and E), chosen to simulate the special pH environment around pancreaticoduodenal anastomoses, which range between 7.15 and 8.93 during the healing stages of chronic wounds. Our results indicated that PVA exhibited lower degradation rates compared with other groups, while the addition of OHAD increased system degradation rates. Furthermore, under simulated pancreatic fluid conditions, significant increases in PVA hydrogel degradation rates were observed (Fig. 3F), whereas other hydrogels showed marginal differences compared with other conditions [18,19]. Degradation is a critical parameter to consider when manufacturing intracorporeal dressings; short-term degradation can prevent complete closure of the anastomotic site, whereas long-term non-degradation can lead to tissue adhesion and biological toxicity. In summary, we found that the degradation rate of hydrogels under high temperature and simulated pancreatic fluid conditions was significantly higher than under other conditions, similar to other studies, and according to this degradation curve, the degradation cycle is consistent with the healing cycle of the pancreaticoduodenal anastomosis [6]. The degradation profile of PC-OHAD@MP aligns with the critical phases of pancreaticoenteric anastomosis healing. As shown in Fig. 3F, under simulated pancreatic fluid (pH 8.0), the hydrogel retained ~60% mass after 14 days, ensuring sustained mechanical support during the early healing phase (1 to 3 weeks) when mechanical integrity is paramount. Subsequent degradation (>20 days) coincides with the transition to tissue remodeling (3 to 6 months), minimizing risks of long-term foreign body reactions. This controlled degradation avoids premature loss of structural support (as seen with rapidly resorbed PGA meshes) while preventing persistent inflammation from non-degradable materials. Such temporal matching is essential for balancing mechanical protection and tissue regeneration in clinical settings.

Severe pancreatic fistula can cause abdominal infection and bleeding, and even develop into severe sepsis [33]. To combat the complex bacterial environment that occurs after abdominal surgery and reduce the emergence of drug-resistant bacteria and the incidence of complications, we loaded MP, which has a broad spectrum of antibacterial activity and can cover the bacteria that proliferate around the pancreaticoduodenal anastomosis, into the hydrogel system [18–20]. To further verify the success of MP loading into the hydrogel and drug release efficiency, we conducted drug release studies [29] (Fig. 3G to I), which showed drug release curves of hydrogels developed under different pH conditions (relationship between cumulative percentage drug release and time). In the experiment for 8 days, with the increase of OHAD concentration, the drug release in the hydrogel increased. Under pH 5.4, 7.4, and simulated pancreatic fluid conditions, the cumulative release of MP loaded in PVA/CS/OHAD hydrogels was greater than 40%, while other group hydrogels had cumulative releases greater than 30%. To elucidate the drug release mechanism, the MP release data were fitted to the Korsmeyer–Peppas model, where *M*_t_/*M*_∞_ is the fractional drug release, *k* is the kinetic constant, and *n* is the diffusional exponent. The model yielded a high correlation coefficient (*R*^2^ = 0.96) with *n* = 0.39 (Fig. S2), indicating a Fickian diffusion-controlled release mechanism [34]. This aligns with the hydrogel’s low swelling ratio (<15%, Fig. 2F), which limits polymer relaxation and swelling-driven drug release. The dominance of diffusion ensures sustained MP delivery, critical for suppressing bacterial colonization during the early postoperative phase. The dominance of diffusion is further supported by the hydrogel’s low swelling ratio, which limits rapid hydration-driven degradation. To enhance the credibility of the conclusions, we cross-validated them by means of first-order kinetics and Higuchi modeling, where the Higuchi model also had a good fit (*R*^2^ = 0.950), which could further confirm the diffusion-dominated mechanism [35]. Therefore, this hydrogel system can effectively load and release MP, serving as a carrier for sustained-release drugs. Interestingly, this phenomenon differs slightly from the swelling curve. Previous studies have shown that the swelling degree of hydrogels decreases with increasing OHAD concentration, possibly because the addition of OHAD helps increase the degree of crosslinking when interacting with other components of the hydrogel, thereby reducing the swelling rate [10,11].

### Excellent in vitro biocompatibility of hydrogels

**Fig. 4.**
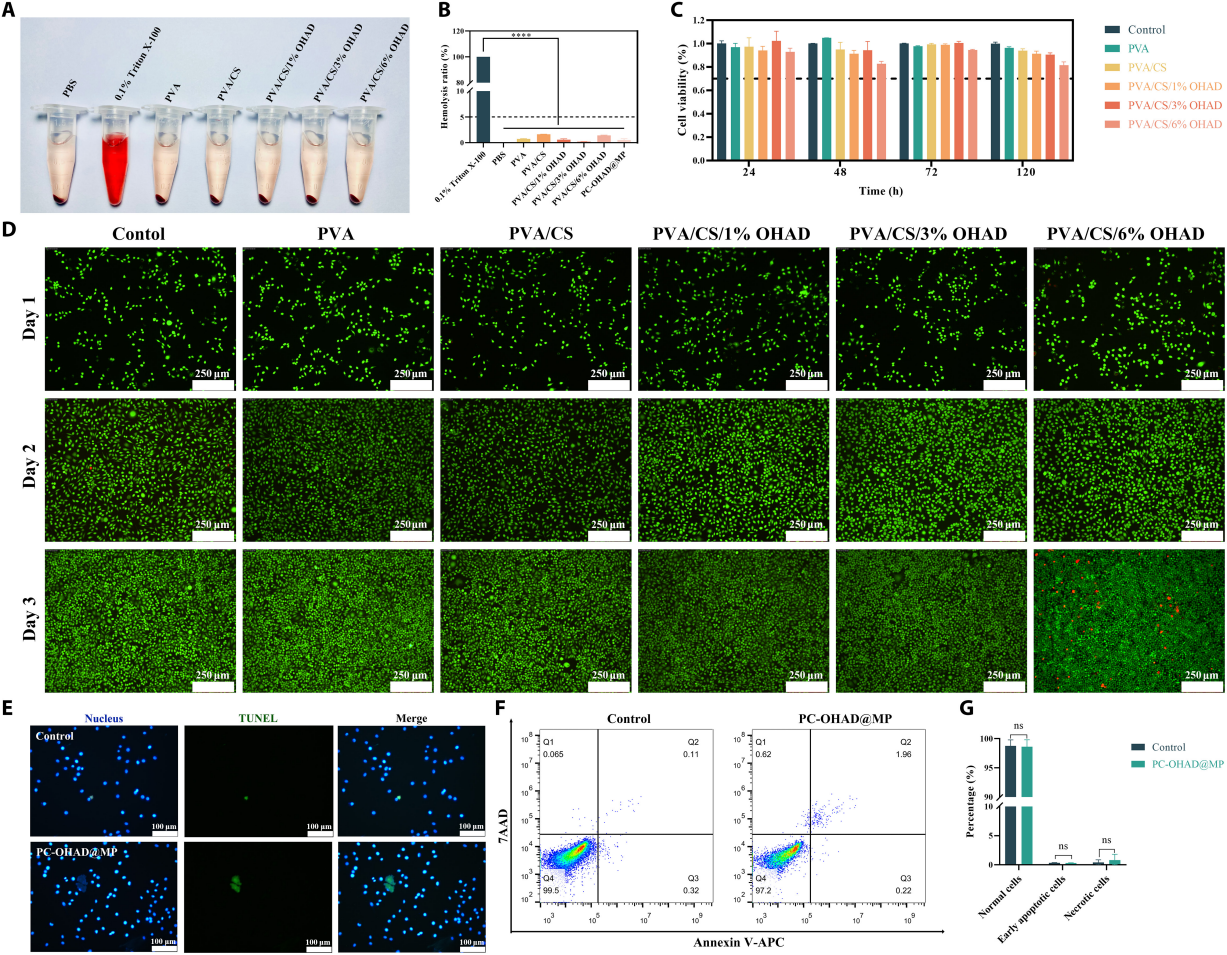
In vitro compatibility of hydrogels. (A) Appearance of hydrogel in vitro hemolysis test (digital camera view) (*n* = 3). (B) Statistical results of hemolysis rate of hydrogels in vitro. (C) Statistical results of cell viability of hydrogel samples with different compositions. (D) Dual staining of live and dead cells under the microscope for hydrogel samples with different composition (*n* = 3). (E) TUNEL staining under the microscope for PC-OHAD@MP hydrogel (*n* = 3). (F) Apoptosis flow cytometry results for PC-OHAD@MP hydrogel (*n* = 3). (G) Statistical results of apoptosis flow cytometry for PC-OHAD@MP hydrogel.

Red blood cells (RBCs) are the most abundant cellular component in the human circulatory system (99%). They are widely used as cellular models for studying the blood compatibility of various compounds, including nanomaterials, in biomedical applications. Therefore, in vitro hemolysis experiments were conducted on PVA, PVA/CS, PVA/CS/1% OHAD, PVA/CS/3% OHAD, and PVA/CS/6% OHAD, as shown in Fig. 4A and Fig. S3A. Similar to the negative control group (PBS), none of these formulations induced hemolysis of RBCs, whereas 0.1% Triton X-100, used as the positive control, clearly caused hemolysis (Fig. 4B). To further validate these results, traditional optical microscopy (Leica, Leica DMi1) was used to observe the morphology of RBCs. Compounds with hemolytic potential, namely, 0.1% Triton X-100, disrupted the integrity of the RBC membrane, leading to hemoglobin release and hemolysis, whereas RBC morphology remained intact in all other groups (Fig. S3B). All experimental results clearly demonstrate that the hydrogels do not affect the molecular structure and permeability of the RBC membrane, that is, that they do not exhibit hemolytic activity.

To observe cell proliferation on the developed hydrogels, L929 cells were cocultured with hydrogel extracts for 5 days. Figure 4C and Fig. S3C demonstrate cell proliferation and viability, indicating that hydrogels are biocompatible with L929 cells. Survival rates of the cells treated with PVA, PVA/CS, and different concentrations of OHAD were all above 70%, meeting the safety grades 0 to 1 according to International Organization for Standardization (ISO) criteria. This confirms that the hydrogels developed in this study are safe. It is noteworthy that although the cell survival rate in the PVA/CS/6% OHAD group was above 70%, it was significantly lower compared with other groups, suggesting that excessively high OHAD concentrations may exhibit some cytotoxicity—a phenomenon consistent with other studies [36]. Figure 4D shows dual staining of live and dead cells over time. The number of cells rapidly increased for PVA, PVA/CS, PVA/CS/1% OHAD, and PVA/CS/3% OHAD, with cells appearing fully viable, consistent with the CCK-8 assay results. Although the number of cells also increased rapidly in the PVA/CS/6% OHAD group, the proportion of red-stained dead cells was significantly higher than that in other groups. Overall, except for PVA/CS/6% OHAD, all hydrogel groups showed no significant cytotoxicity.

In summary, the optimal concentration chosen was PVA/CS/3% OHAD, which was used to synthesize the final hydrogel loaded with MP, named PC-OHAD@MP. To further evaluate whether PC-OHAD@MP induces apoptosis, TUNEL staining was performed and showed results similar to the control group, indicating no significant apoptosis (Fig. 4E). Flow cytometry apoptosis detection also revealed no significant differences in the proportions of normal cells, early apoptotic cells, and necrotic cells between the PC-OHAD@MP and control groups (Fig. 4F and G). These results collectively demonstrate that the hydrogel exhibits excellent in vitro biocompatibility, providing a solid preliminary basis for its application in vivo.

### Hydrogel promotes fibroblast proliferation and migration in vitro

**Fig. 5.**
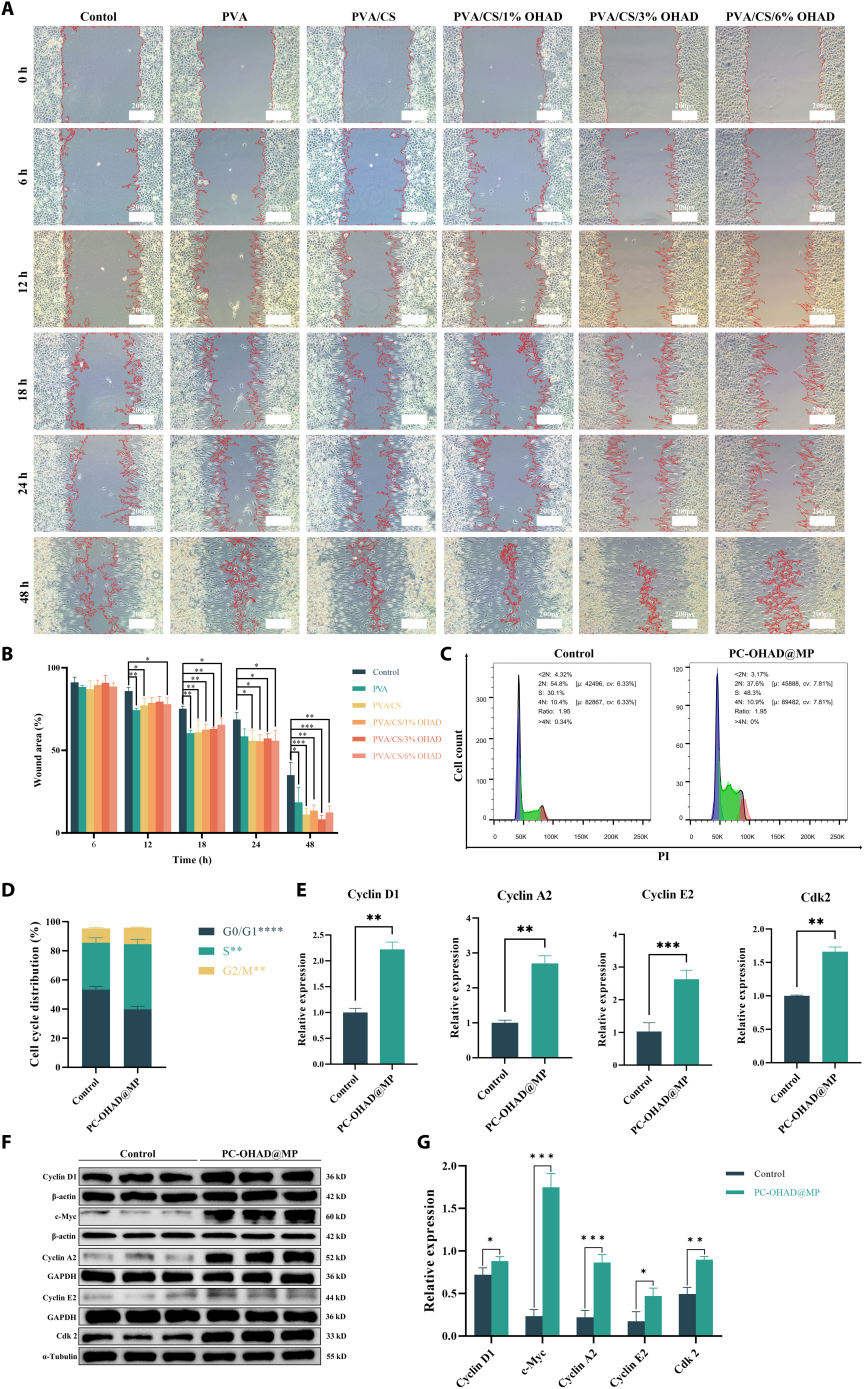
Hydrogel enhances cell proliferation and migration. (A) Scratch assay under the microscope for hydrogel samples with different compositions (*n* = 3). (B) Statistical results of scratch assay for hydrogel samples with different compositions. (C) Cell cycle flow cytometry results for PC-OHAD@MP hydrogel (*n* = 3). (D) Statistical results of cell cycle apoptosis for PC-OHAD@MP hydrogel. (E) Relative mRNA expression levels of key regulatory genes in the cell cycle for PC-OHAD@MP hydrogel (*n* = 3). (F) Quantitative analysis results of protein expression levels of key regulatory genes in the cell cycle for PC-OHAD@MP hydrogel (*n* = 3). (G) Statistical results of protein expression levels of key regulatory genes in the cell cycle for PC-OHAD@MP hydrogel.

Scratch assays were utilized to assess the effects of PVA, PVA/CS, PVA/CS/1% OHAD, PVA/CS/3% OHAD, and PVA/CS/6% OHAD solutions on cell proliferation and migration. The results demonstrated that compared with the untreated group, all formulations of PVA, PVA/CS, PVA/CS/1% OHAD, PVA/CS/3% OHAD, and PVA/CS/6% OHAD enhanced cell migration and proliferation (Fig. 5A). Among them, PVA/CS/3% OHAD showed the most significant effect, reducing the remaining area by 1.2-fold after 24 h and by 4.22-fold after 48 h compared with the control group (Fig. 5B). We did not perform scratch experiments with the PC-OHAD@MP group, but the healing-promoting properties of hydrogels are mainly derived from their polymer composition. The degradation products of PC-OHAD@MP, including CS oligosaccharides and dopamine metabolites, are critical to its long-term biocompatibility and anti-inflammatory effects. CS oligosaccharides, generated during hydrogel degradation, have been shown to suppress NF-κB signaling by inhibiting IKKβ phosphorylation, thereby reducing pro-inflammatory cytokine production (e.g., tumor necrosis factor-α [TNF-α] and interleukin-1β [IL-1β]). Concurrently, dopamine metabolites (e.g., 5,6-dihydroxyindole) exhibit antioxidant properties, scavenging reactive oxygen species that exacerbate pancreatic inflammation [14]. These bioactive degradation products synergistically modulate the wound microenvironment, transitioning it from a pro-inflammatory to a prohealing state. These findings are consistent with previous studies indicating that PVA, CS, and OHAD promote cell proliferation and migration, which can facilitate wound healing [37]. A combination of Transwell (Fig. S4) and cell scratch assay (Fig. S5) results confirmed that these effects are inherent to the hydrogel matrix and are not related to antibiotic release. The partitioning of these 2 functions, the structural/biological support of hydrogels and the infection control of MP, ensures a balanced therapeutic strategy for the prevention of POPF. Furthermore, the cell cycle analysis revealed that compared with the control group, PC-OHAD@MP-treated cells exhibited a significant decrease in G0/G1-phase cells and an increase in S-phase cells, with a similar percentage of cells in the G2/M phase (Fig. 5C and D). To further elucidate the specific changes in the cell cycle, quantitative polymerase chain reaction (qPCR) was employed to validate the expression of key genes involved in the cell cycle, including Cyclin D1, Cyclin A2, Cyclin E2, and Cdk2 [38]. As shown in Fig. 5E, PC-OHAD@MP treatment up-regulated the expression of Cyclin D1, Cyclin A2, Cyclin E2, and Cdk2 genes. Similarly, Western blot analysis showed statistically significant up-regulation of Cyclin D1, Cyclin A2, Cyclin E2, Cdk2, and the key transcription factor c-Myc protein levels in PC-OHAD@MP-treated cells (Fig. 5F and G). These results indicate that the hydrogel treatment significantly enhances cell proliferation and migration, likely through activation of the cell cycle [39]. These results demonstrate that the hydrogel treatment significantly promotes cell proliferation and migration, likely through the activation of the cell cycle. This observation is consistent with our previous studies and may be attributed to the unique structural characteristics of CS as a polysaccharide, which exhibits functional properties similar to those of glycosaminoglycans [40]. As key components of the extracellular matrix, glycosaminoglycans play critical roles in cell proliferation, differentiation, and morphogenesis. Given its polysaccharide nature, CS also exhibits similar effects, promoting cellular activities such as proliferation and migration, which are crucial for tissue repair and regeneration. In summary, the developed hydrogel formulation, particularly PC-OHAD@MP, not only promotes fibroblast proliferation and migration in vitro, but also induces changes in the cell cycle regulatory pathways that are crucial for wound healing processes. These findings underscore the potential of the PC-OHAD@MP hydrogel as an effective therapeutic agent for promoting tissue regeneration and wound healing applications.

### PC-OHAD@MP exhibits strong in vitro antibacterial activity

**Fig. 6.**
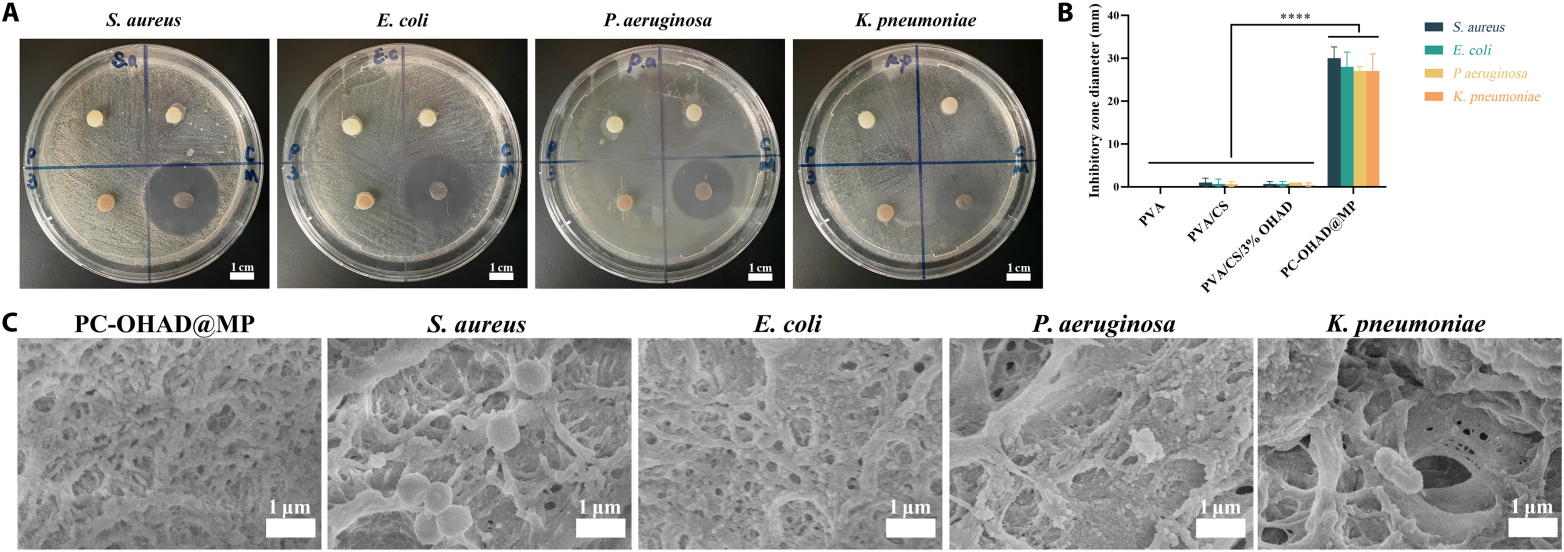
Hydrogel exhibits strong antibacterial activity. (A) Appearance of inhibition zones in agar diffusion assay under a digital camera for PVA, PVA/CS, PVA/CS/3% OHAD, and PC-OHAD@MP hydrogels against *Staphylococcus aureus* (*S. aureus*), *Escherichia coli* (*E. coli*), *Pseudomonas aeruginosa* (*P. aeruginosa*), and *Klebsiella pneumoniae* (*K. pneumoniae*) (*n* = 3). (B) Statistical results of inhibition zones in agar diffusion assay. (C) Coculture of PC-OHAD@MP and PVA/CS/3% OHAD hydrogels with *S. aureus*, *E. coli*, *P. aeruginosa*, and *K. pneumoniae* under an electron microscope.

Intraperitoneal bacterial contamination is a risk factor for pancreatic fistula, and bacterial infections often hinder anastomotic healing [18]. Although CS provides the hydrogel with intrinsic antimicrobial activity and wound healing support, its antimicrobial activity is not sufficient to resist the complex bacterial environment of the abdominal cavity, and to enhance the antimicrobial activity, an MP-loaded hydrogel was used. MP was chosen because of its broad-spectrum antimicrobial activity against *Enterococcus* and *P. aeruginosa*, the main pathogens of POPF. MP functions by penetrating the cell wall of both gram-positive and gram-negative bacteria to reach their target penicillin-binding proteins, thereby inhibiting cell-wall synthesis and exerting antibacterial effects [21,41]. The antibacterial activity of PVA, PVA/CS, PVA/CS/3% OHAD, and PC-OHAD@MP against *S. aureus*, *E. coli*, *P. aeruginosa*, and *K. pneumoniae* was evaluated. The results showed that PC-OHAD@MP significantly inhibited all 4 bacterial strains compared with the other 3 non-drug-loaded hydrogel groups. The number of bacteria around the hydrogel was markedly reduced, with inhibition zone diameters of 30.00 ± 2.65 mm for *S. aureus*, 28.00 ± 3.46 mm for *E. coli*, 27.00 ± 1.00 mm for *P. aeruginosa*, and 27.00 ± 4.00 mm for *K. pneumoniae* (Fig. 6A and B). Further SEM analysis revealed almost no bacterial adhesion on the surface of PC-OHAD@MP compared with the non-drug-loaded hydrogels (Fig. 6C). These results clearly demonstrate that the PC-OHAD@MP hydrogel exhibits potent antibacterial properties, consistent with previous reports [29]. In conclusion, PC-OHAD@MP shows broad-spectrum antibacterial activity against most bacteria that can cause intraperitoneal infections and POPFs. This suggests its potential application for preventing and treating POPFs and intraperitoneal bacterial infections.

### PC-OHAD@MP effectively reduces pancreatic fistula formation in rats and demonstrates good in vivo biocompatibility

**Fig. 7.**
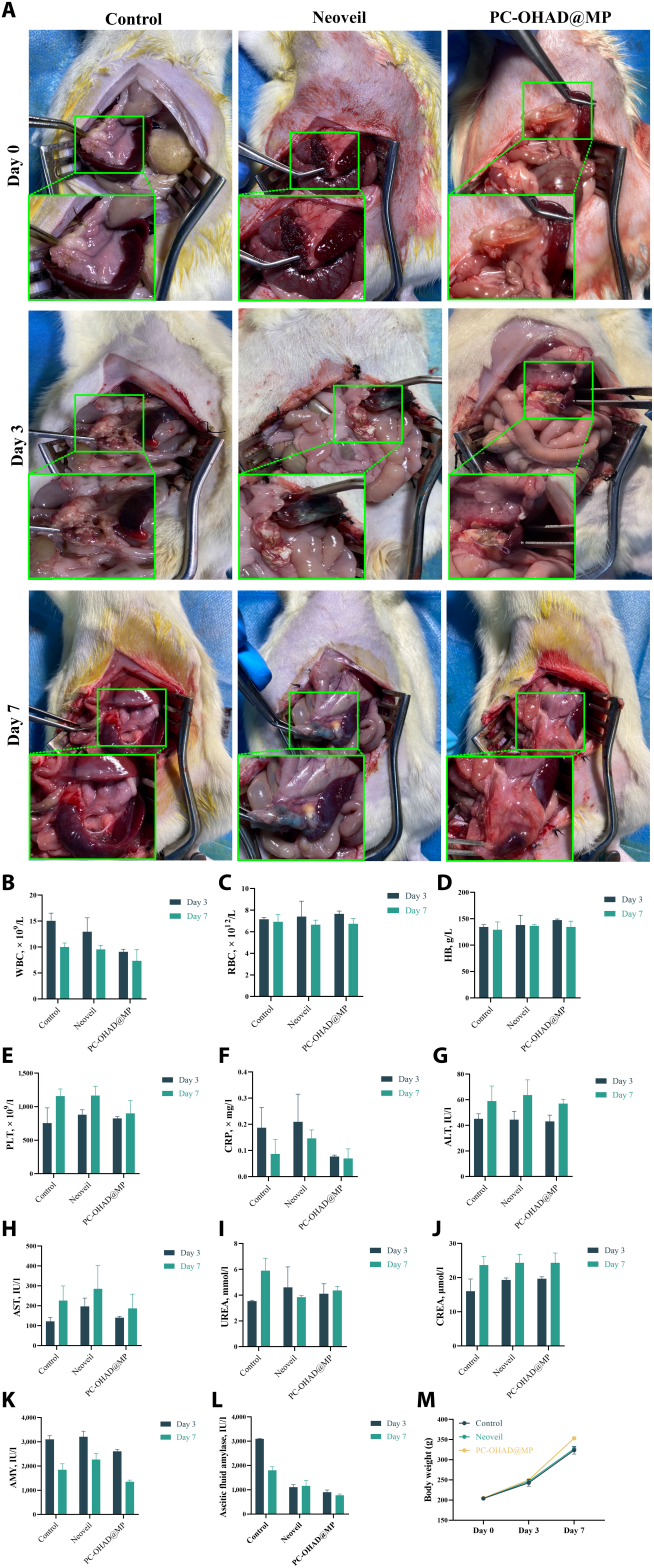
In vivo biocompatibility of hydrogel and reduction of postoperative pancreatic fistula. (A) Appearance of surgical sites at postoperative days 0, 3, and 7 under a digital camera for the control group, Neoveil, and PC-OHAD@MP (*n* = 5). (B to L) Blood routine tests, CRP, ALT, AST, urea, CREA, blood amylase, and abdominal amylase levels at postoperative days 0, 3, and 7 for the control group, Neoveil, and PC-OHAD@MP (*n* = 5). (M) Preoperative weight gain curve at postoperative days 0, 3, and 7 for the control group, Neoveil, and PC-OHAD@MP (*n* = 5).

Significantly elevated pancreatic enzyme levels in abdominal fluid indicate the formation of pancreatic fistula (Fig. 7L). As shown in Fig. 7A, on postoperative days 3 and 7, Neoveil and PC-OHAD@MP were enveloped by surrounding tissues, reducing pancreatic exposure, whereas the control group showed less envelopment, with exposed suture sites and areas of pancreatic necrosis and saponification spots. Blood routine and biochemical results depicted in Fig. 7B to J revealed no significant differences in RBC, hemoglobin (HB), platelets (PLT), ALT, AST, urea, and CREA among the groups, further indicating that Neoveil and PC-OHAD@MP possess good in vivo compatibility. However, white blood cell and CRP levels in the PC-OHAD@MP group were lower than those in the other 2 groups, suggesting its potent anti-inflammatory and antibacterial effects. Similarly, as shown in Fig. 7K, both blood and abdominal amylase levels were lower in the PC-OHAD@MP group compared with the other groups, indicating its role in alleviating pancreatic inflammation and reducing POPF. The rats demonstrated a gradual increase in body weight pre- and postoperation, and no significant changes in appetite were observed with surgery, Neoveil, and PC-OHAD@MP (Fig. 7M), further confirming that PC-OHAD@MP has no significant toxicity and exhibits excellent in vivo biocompatibility. While the current study focuses on the integrated PC-OHAD@MP system, existing literature provides insights into the individual contributions of its components. CS is well documented to enhance wound healing through direct antibacterial activity and fibroblast stimulation. OHAD promotes cell adhesion and reduces oxidative stress via catechol-mediated interactions. The PVA matrix ensures mechanical stability critical for surgical handling. Although a non-drug-loaded PC-OHAD control group was not included, prior studies on similar hydrogels confirm that CS and OHAD synergistically improve anastomotic healing independent of antibiotics [10,14,16]. The addition of MP specifically targets bacterial contamination, addressing a key risk factor for POPF. While a non-drug-loaded hydrogel group would further isolate component effects, our primary aim was to evaluate the hydrogel as a multifunctional system. Prior studies confirm that CS and OHAD alone reduce inflammation and promote fibroblast migration, supporting the design choice to focus on the integrated formulation. The rat pancreatic duct transection model was selected based on its use in evaluating pancreatic fistula interventions [25,26]. While it does not replicate the full PD procedure, it balances ethical considerations and technical feasibility for preliminary validation. However, we must recognize the shortcomings of the pancreatic fistula rat model in that it does not fully replicate the complexities of human PD. Clinical PD involves pancreatico-intestinal anastomosis under mechanical stress, enzyme exposure, and revascularization, none of which were modeled here. However, this simplified model is consistent with established protocols for initial screening of pancreatic closure materials, focusing on sealing effectiveness and acute inflammatory response. Future long-term follow-up (>30 days) studies in large animals (e.g., porcine pancreatic prosthesis model) will evaluate the performance of hydrogels under clinically relevant conditions, including anastomotic healing, fibrosis, and pancreatic enzyme activity [6].

**Fig. 8.**
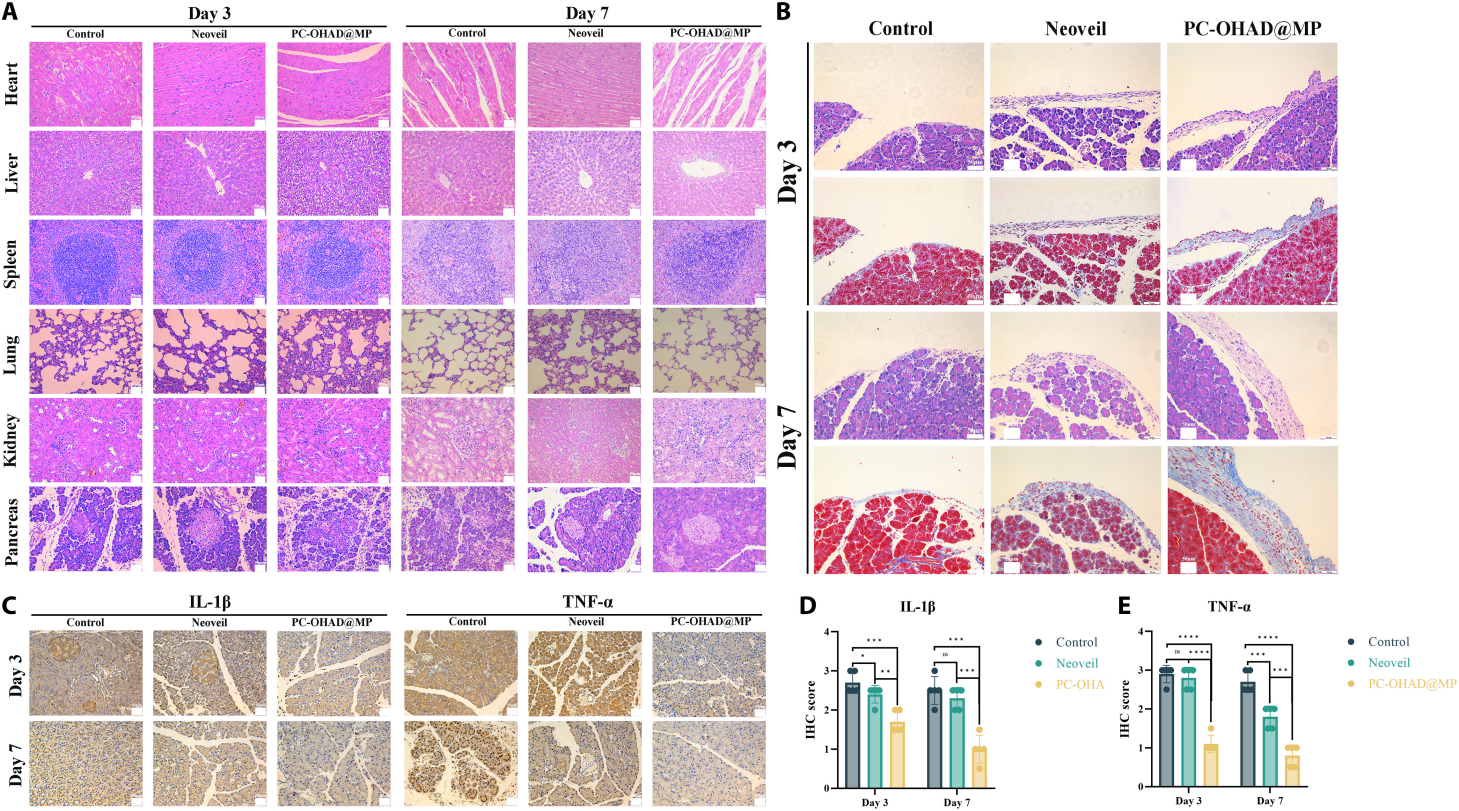
In vivo biocompatibility, promotion of collagen fiber deposition, and anti-inflammatory capability of PC-OHAD@MP. (A) Histological examination of heart, liver, spleen, lung, kidney, and pancreatic tissues stained with H&E at postoperative days 3 and 7 for the control group, Neoveil, and PC-OHAD@MP (*n* = 5). (B) Masson’s trichrome staining of pancreatic tissues at suture sites at postoperative days 3 and 7 for the control group, Neoveil, and PC-OHAD@MP (*n* = 5). (C) Immunohistochemical staining of IL-1β and TNF-α levels in pancreatic tissues at suture sites at postoperative days 3 and 7 for the control group, Neoveil, and PC-OHAD@MP (*n* = 5). (D) Statistical results of immunohistochemical staining for IL-1β levels in pancreatic tissues at suture sites at postoperative days 3 and 7 for the control group, Neoveil, and PC-OHAD@MP. (E) Statistical results of immunohistochemical staining for TNF-α levels in pancreatic tissues at suture sites at postoperative days 3 and 7 for the control group, Neoveil, and PC-OHAD@MP.

To further validate the in vivo compatibility, collagen fiber deposition capability, and anti-inflammatory properties of PC-OHAD@MP, we collected organ and suture tissue samples on postoperative days 3 and 7 for hematoxylin and eosin (H&E) staining and Masson’s trichrome staining. The results from H&E staining (Fig. 8A) demonstrated no significant damage to organs such as the heart, liver, spleen, lungs, kidneys, and pancreas by PC-OHAD@MP. Masson’s staining (Fig. 8B) revealed dense collagen deposition with PC-OHAD@MP. Despite the hydrogel’s synthetic components, histopathological analysis revealed no significant immune cell infiltration (e.g., neutrophils and macrophages) at the anastomotic site, suggesting low immunogenicity. This may be attributed to the biocompatibility of PVA and CS, which are Food and Drug Administration-approved for medical devices. Furthermore, dopamine modification minimizes surface irritation by mimicking endogenous catecholamine adhesion mechanisms. Immunohistochemical staining for IL-1β and TNF-α (Fig. 8C) showed pronounced brown-yellow staining in the control group, slightly lighter staining in the Neoveil group compared with the control, and significantly reduced brown-yellow staining in the PC-OHAD@MP group [42]. Quantitative scoring of the immunohistochemical staining intensity across the groups (Fig. 8D and E) indicated that PC-OHAD@MP significantly lowered IL-1β and TNF-α levels. IL-1β and TNF-α are crucial inflammatory cytokines, with TNF-α being the earliest and most important inflammatory mediator in the inflammatory response process, capable of activating neutrophils and lymphocytes, increasing endothelial cell permeability, regulating metabolic activity in other tissues, and promoting synthesis and release of other cytokines. IL-1β is a typical proinflammatory cytokine that induces the release of inflammatory cytokines such as IL-6 and TNF-α, stimulating T-cell activation and initiating local and systemic inflammatory responses [43]. These chemical staining results demonstrate that PC-OHAD@MP possesses potent anti-inflammatory effects. Although our study focused on short-term results (7 days), the controlled degradation of PC-OHAD@MP demonstrated minimal long-term residue in pancreatic tissue. Histopathological analysis showed that the degradation products did not induce chronic inflammation. Previous studies on CS hydrogels have confirmed that oligosaccharides are cleared by renal excretion, whereas dopamine derivatives are metabolized via the endogenous catecholamine pathway. To further validate long-term safety, future studies will monitor pancreatic enzyme levels (e.g., amylase and lipase) and endocrine function (e.g., insulin secretion) for 3 to 6 months in a large-animal model.

In summary, PC-OHAD@MP accelerates closure of suture sites, promotes collagen deposition, mitigates pancreatic inflammation, and does not induce severe immune-inflammatory reactions. It effectively reduces pancreatic fistula formation in rats and exhibits excellent in vivo biocompatibility.

### PC-OHAD@MP reduces tissue inflammation by inhibiting the NF-κB signaling pathway

**Fig. 9.**
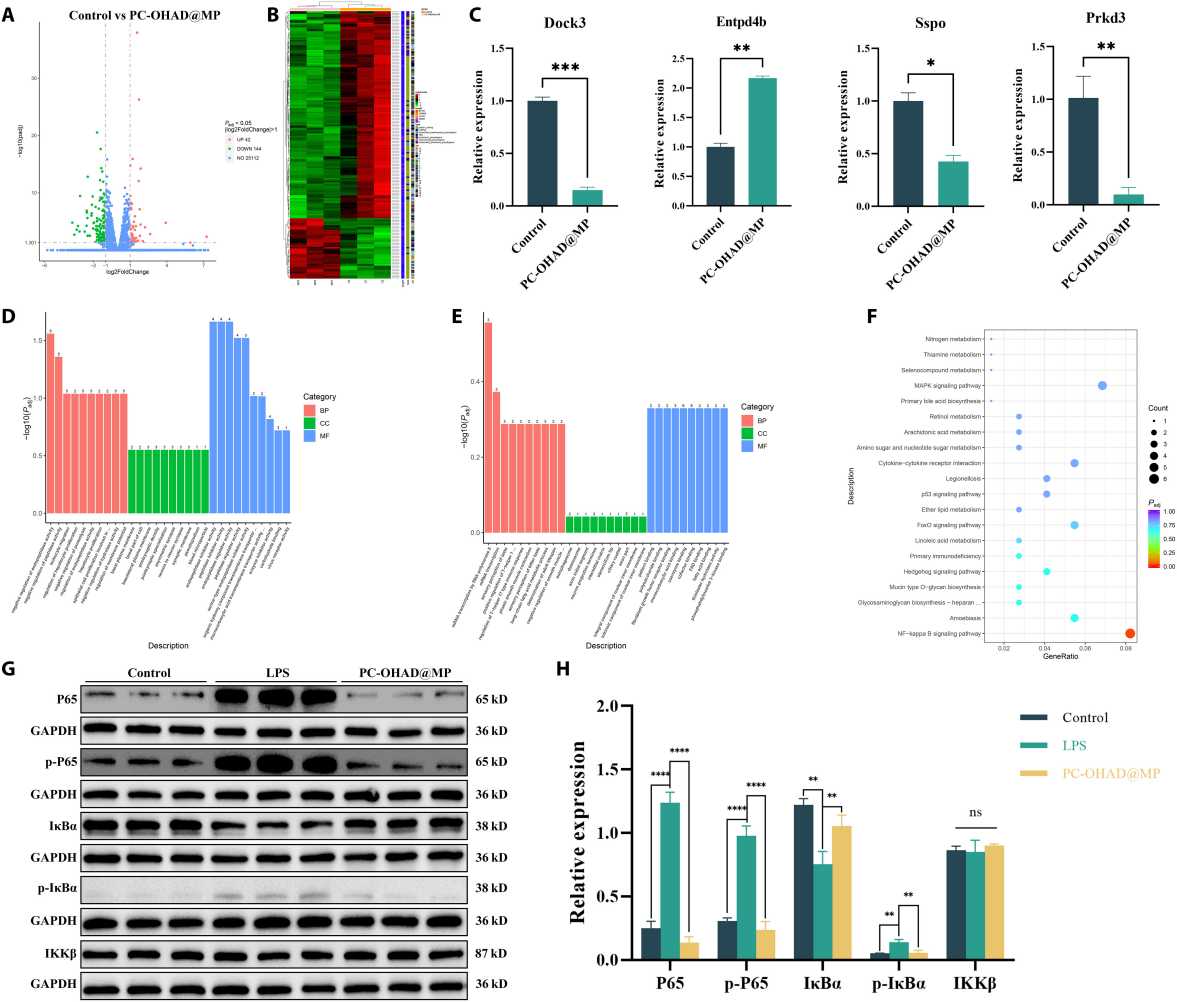
Hydrogel promotes cell proliferation, migration, and anti-inflammatory mechanisms. (A) Volcano plot showing differentially expressed genes between the control group and the PC-OHAD@MP group (*n* = 3). (B) Heatmap displaying differentially expressed genes between the control group and the PC-OHAD@MP group (*n* = 3). (C) Relative mRNA expression levels of Dock3, Entpd4b, Sspo, and Prkd3 between the control group and the PC-OHAD@MP group (*n* = 3). (D) Up-regulated Gene Ontology (GO) enrichment analysis results between the control group and the PC-OHAD@MP group (*n* = 3). (E) Down-regulated GO enrichment analysis results between the control group and the PC-OHAD@MP group (*n* = 3). (F) Kyoto Encyclopedia of Genes and Genomes (KEGG) enrichment analysis results between the control group and the PC-OHAD@MP group (*n* = 3). (G) Protein expression levels of key genes in the NF-κB pathway (P65, p-P65, p-IκBα, IκBα, and IKKβ) between the control group and the PC-OHAD@MP group (*n* = 3). (H) Quantitative analysis of protein expression levels of key genes in the NF-κB pathway (P65, p-P65, p-IκBα, IκBα, and IKKβ) between the control group and the PC-OHAD@MP group.

To further explore the underlying mechanisms through which PC-OHAD@MP exerts its effects, we conducted transcriptome analysis using samples collected from the control and PC-OHAD@MP groups. The results revealed significant differences in gene expression, with volcano plots and heatmaps showing 42 significantly up-regulated genes and 144 significantly down-regulated genes (Fig. 9A and B). To validate the reliability of the sequencing results, we screened the genes with significant differences, identifying 9 genes with log_2_|FoldChange| > 3 (Table S1). qPCR validation of Dock3, Entpd4b, Sspo, and Prkd3 genes confirmed consistency with the sequencing results, showing increased mRNA expression for Dock3, Sspo, and Prkd3, and decreased expression for Entpd4b (Fig. 9C). This confirmed the reliability of the sequencing results, with these genes known from previous studies to affect cell proliferation and migration [44–46]. Compared to peptide-modified hydrogels relying on costly synthetic ligands, our OHAD modification utilizes a bioinspired, cost-effective approach to enhance cell–matrix interactions. Gene Ontology (GO) database analysis revealed that the genes showing expression changes are involved in crucial biological processes (BP), cellular components (CC), and molecular functions (MF) related to tissue healing and organismal inflammation regulation (Fig. 9D and E). Furthermore, Kyoto Encyclopedia of Genes and Genomes (KEGG) analysis explored potential signaling pathways. As shown in Fig. 9F, the down-regulated genes in the PC-OHAD@MP group were predominantly enriched in the NF-κB signaling pathway. This suggests that the PC-OHAD@MP hydrogel may reduce the expression of inflammatory cytokines by inhibiting the NF-κB signaling pathway. This pathway is well-known for its critical role in inflammation regulation [47]. To validate these results, Western blot analysis was performed on the key genes involved in this pathway. To simulate in vivo macrophage activation, RAW264.7 macrophages were first induced with lipopolysaccharide (LPS) to produce a series of inflammatory cytokines, followed by treatment with PC-OHAD@MP [22]. Compared with the LPS-treated group, the PC-OHAD@MP hydrogel-treated group showed significant reductions in P65, p-P65, and p-IκBα levels, along with an increase in IκBα levels (Fig. 9G and H). This confirms that PC-OHAD@MP attenuates the expression of inflammatory cytokines in macrophages through the NF-κB pathway, consistent with the down-regulation of proinflammatory cytokines such as IL-6 and TNF-α observed in immunohistochemistry. In summary, these findings demonstrate that PC-OHAD@MP accelerates wound healing at suture sites by promoting cell proliferation and migration, and regulating proinflammatory pathways. This mechanism reduces the occurrence of POPF.

## Conclusion

In this study, we developed a multifunctional hydrogel with efficient antimicrobial, anti-inflammatory, and wound healing properties. The hydrogel is based on a composition of PVA, CS, and OHAD, and has excellent plasticity, easy clinical applicability, good biocompatibility, moderate mechanical strength, low swelling, and strong adhesion. These characteristics meet the requirements of the surgical site and its surroundings. The hydrogel is also anti-inflammatory and antimicrobial and promotes wound healing at the anastomosis site, which ultimately leads to closure of the pancreaticoenteric anastomosis and the reduction of POPF. While PC-OHAD@MP demonstrates promising multifunctionality in preclinical models, further optimization and validation in large-animal studies are essential before clinical translation. Our findings provide a foundational framework for developing next-generation biomaterials to address POPF.

## Data Availability

The data that support the findings of this study are available from the corresponding authors upon reasonable request.
