# Supplementary material for: Multiaction Antimicrobial, Anti-inflammatory, and Prohealing Hydrogel as a Novel Strategy for Preventing Postoperative Pancreatic Fistula
Source: Biomater Res. 2025 Apr 23;29:0194. doi: 10.34133/bmr.0194 (PMC12015097; doi:10.34133/bmr.0194)
Supplement: Supplementary 1 — Figs. S1 to S5 Table S1 [file bmr.0194.f1.docx]

**Supplementary Materials**

Multi-action antimicrobial, anti-inflammatory, and pro-healing hydrogel as a new strategy for preventing postoperative pancreatic fistula

**Supplement-Materials and Result**

**S-1 Transcriptome sequencing.**

**Table S1** **List of genes with log_2_|FoldChange| > 3 in the sequencing results.**

| gene_id | log_2_FoldChange | pvalue | padj | gene_name | gene_description |
| --- | --- | --- | --- | --- | --- |
| ENSMUSG00000063286 | -3.067333813 | 8.05E-13 | 3.38E-10 | Gvin-ps7 | GTPase, very large interferon inducible, pseudogene 7 |
| ENSMUSG00000039716 | -3.703534779 | 2.49E-07 | 1.64E-05 | Dock3 | dedicator of cyto-kinesis 3 |
| ENSMUSG00000022066 | 3.904341311 | 2.65E-07 | 1.73E-05 | Entpd4b | ectonucleoside triphosphate diphosphohydrolase 4B |
| ENSMUSG00000107660 | -3.235143814 | 1.16E-06 | 6.07E-05 | Gm6559 | predicted gene 6559 |
| ENSMUSG00000029797 | -3.342226624 | 7.75E-06 | 0.000269368 | Sspo | SCO-spondin |
| ENSMUSG00000024070 | -3.118450785 | 1.88E-05 | 0.000545075 | Prkd3 | protein kinase D3 |
| ENSMUSG00000078607 | -3.535224399 | 9.77E-05 | 0.001986908 | 1810010H24Rik | RIKEN cDNA 1810010H24 gene |
| ENSMUSG00000060256 | 7.238313895 | 0.000288607 | 0.004650241 | Tdpoz4 | TD and POZ domain containing 4 |
| ENSMUSG00000096175 | 5.902543432 | 0.006985567 | 0.049380076 | Gm21761 | predicted gene, 21761 |

**S-2 Hydrogel has good elasticity**

As shown in ***Fig S1***, the hydrogel we developed has good elasticity and can quickly return to its original state after compression.


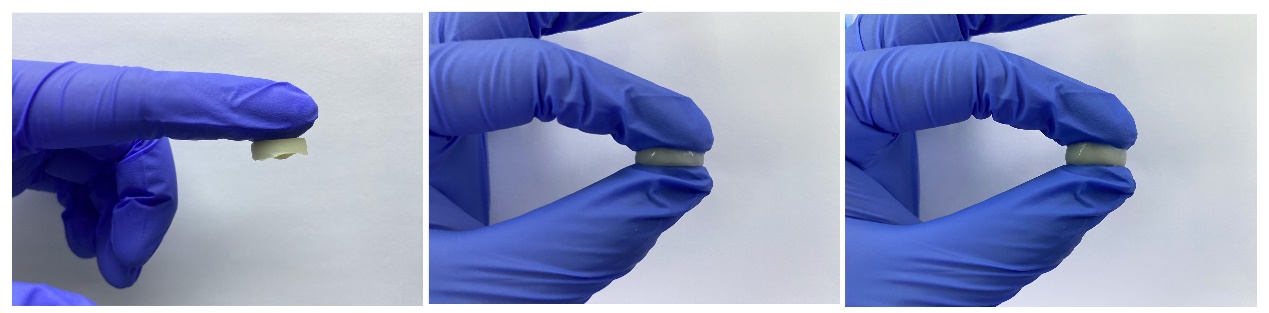


***Fig S1.*** *Hydrogel extrusion performance test.*

**S-3 Meropenem release mechanism**

As shown in ***Fig S2***, to elucidate the drug release mechanism, the PC-OHAD hydrogel release data of meropenem were fitted to three drug release models. As can be seen from ***Fig S2C***, the correlation coefficient of the fitted Korsmeyer-Peppas model was high (R^2^ = 0.96) with n = 0.39, suggesting that this is a Fickian diffusion-controlled release mechanism. ***Fig*** ***S2A*** and ***2B*** were cross-validated by first-order kinetics and Higuchi modeling, which was also well fitted (𝑅^2^ = 0.950 ), which further confirmed the diffusion-dominated mechanism.


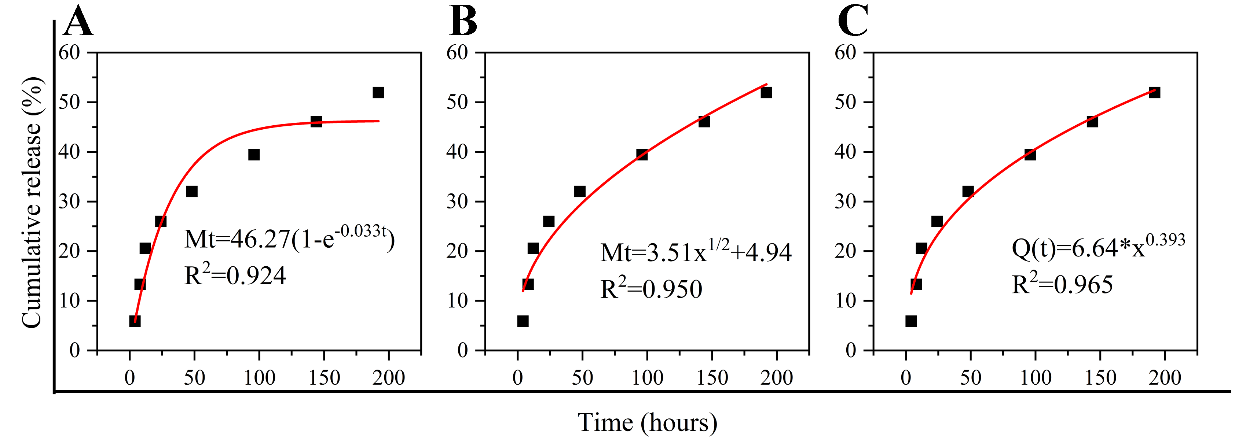


***Fig S2****. PC-OHAD hydrogel release of meropenem was fitted to the three release models. (A) First-order kinetics modeling. (B) Higuchi modeling. (C) Korsmeyer-Peppas model.*

**S-4 The hydrogel has good biocompatibility.**

As shown in ***Fig S3A***, each hydrogel group similarly to the negative control group does not cause hemolysis of erythrocytes, whereas the 0.1% Triton X-100 group showed significant hemolysis of erythrocytes. Similarly, the microscopic view in ***Fig S3B*** shows that the RBCs in the 0.1% Triton X-100 group sent hemolyzed and ruptured, and the erythrocyte morphology disappeared, whereas the RBCs in the other groups maintained their basic morphology. From ***Fig S3C*** it can be noticed that hydrogel similar to control group does not affect the level of cell proliferation.


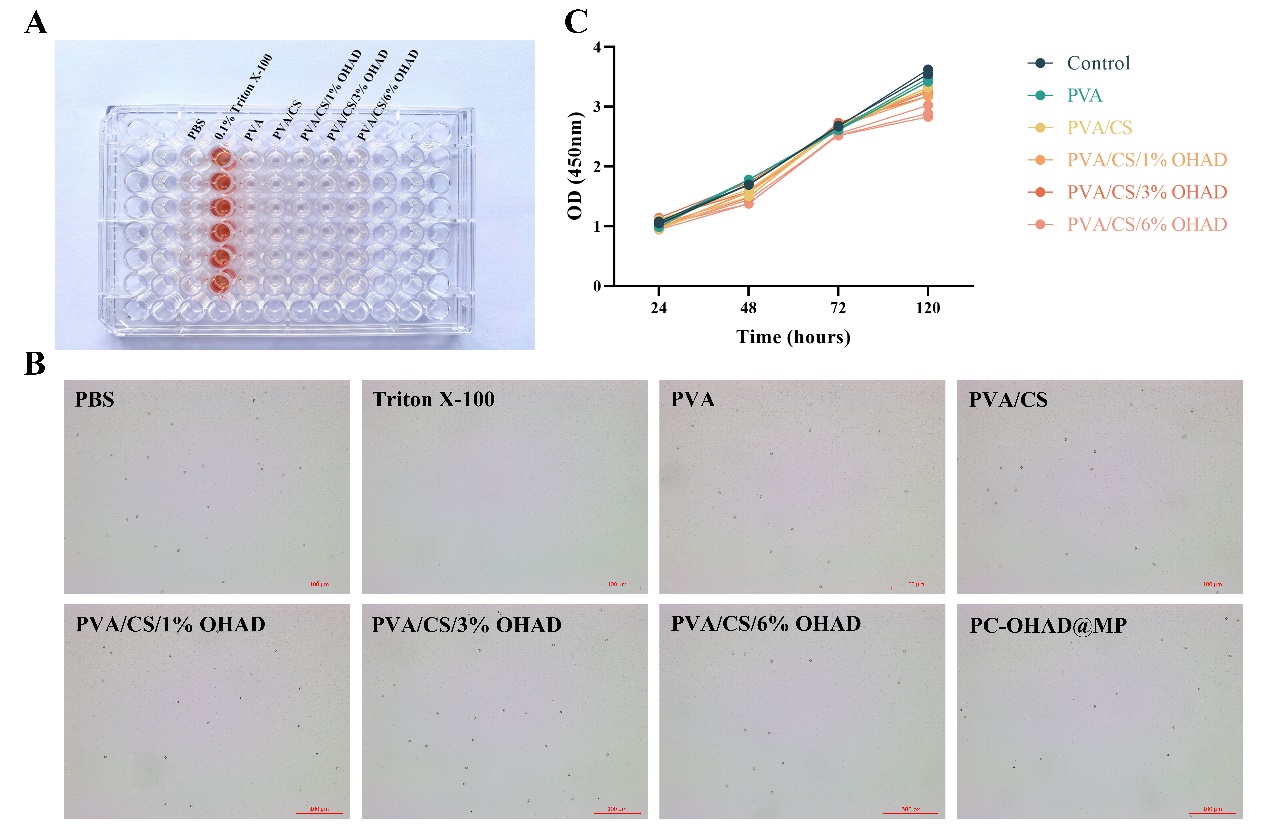


***Fig S3.*** *In vitro compatibility of hydrogels. (A) Appearance of the supernatant of an in vitro hemolysis test centrifuge tube placed in a 96-well plate (digital camera view) (n = 6). (B) PBS, 0.1% Triton X-100 with hydrogel RBC microscopic view (n = 6). (C) Cell proliferation curves for each hydrogel group (n = 3).*

**S-5 PVA/CS/3% OHAD hydrogel promotes cell migration.**

Transwell experiments were utilized to further assess whether each hydrogel promoted cell migration. As can be seen from ***Fig S4***, there was some promotion of cell migration in the PVACS group compared to the Control and PVA groups, which was related to the incorporation of CS. The cell migration rate of the PVA/CS/3% OHAD and PC-OHAD groups was significantly better than that of the other groups, indicating that the hydrogel of this system could promote cell migration, while the addition of MP did not affect cell migration.

**
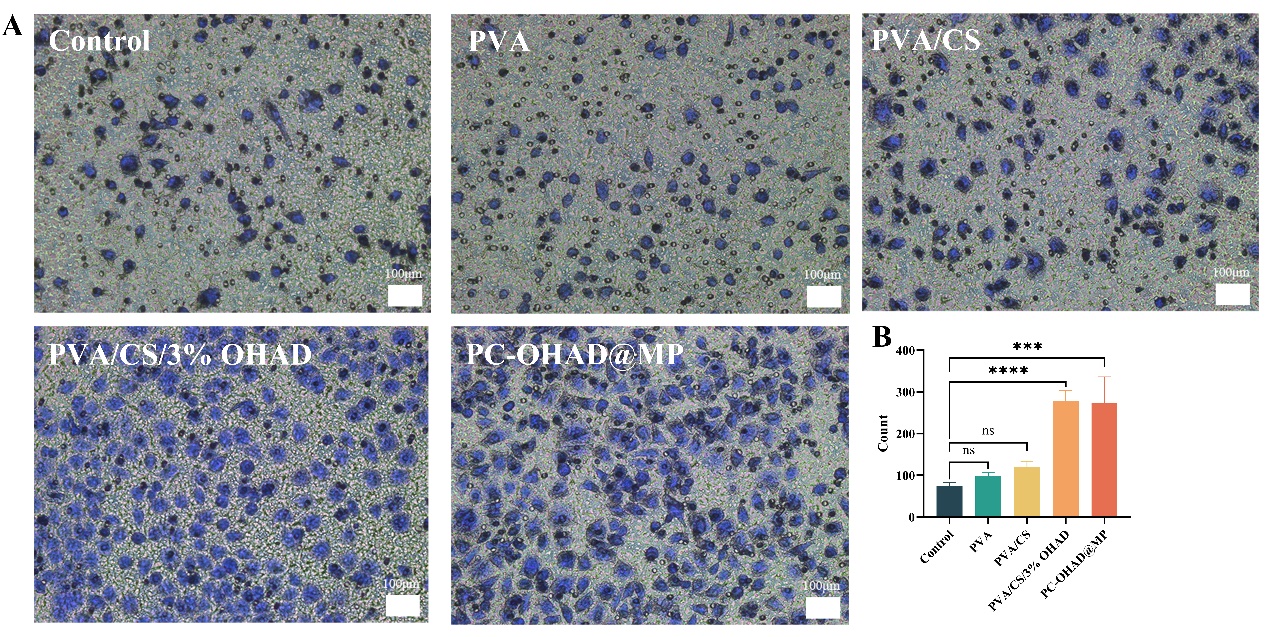
**

***Fig S4****. (A) Transwell experiment results (n = 3). (B) Statistical results of the Transwell experiment.*

**S-6 PVA/CS/3% OHAD hydrogel promotes cell adhesion.**

Focal adhesion staining (Vinculin immunofluorescence) was performed on L929 cells to compare the adhesion differences between the PC-OHAD@MP group and the unmodified hydrogel group. As shown in ***Fig S5***, the expression of Vinculin in PC-OHAD@MP and its carrier PVA/CS/3% OHAD was higher than the other groups.


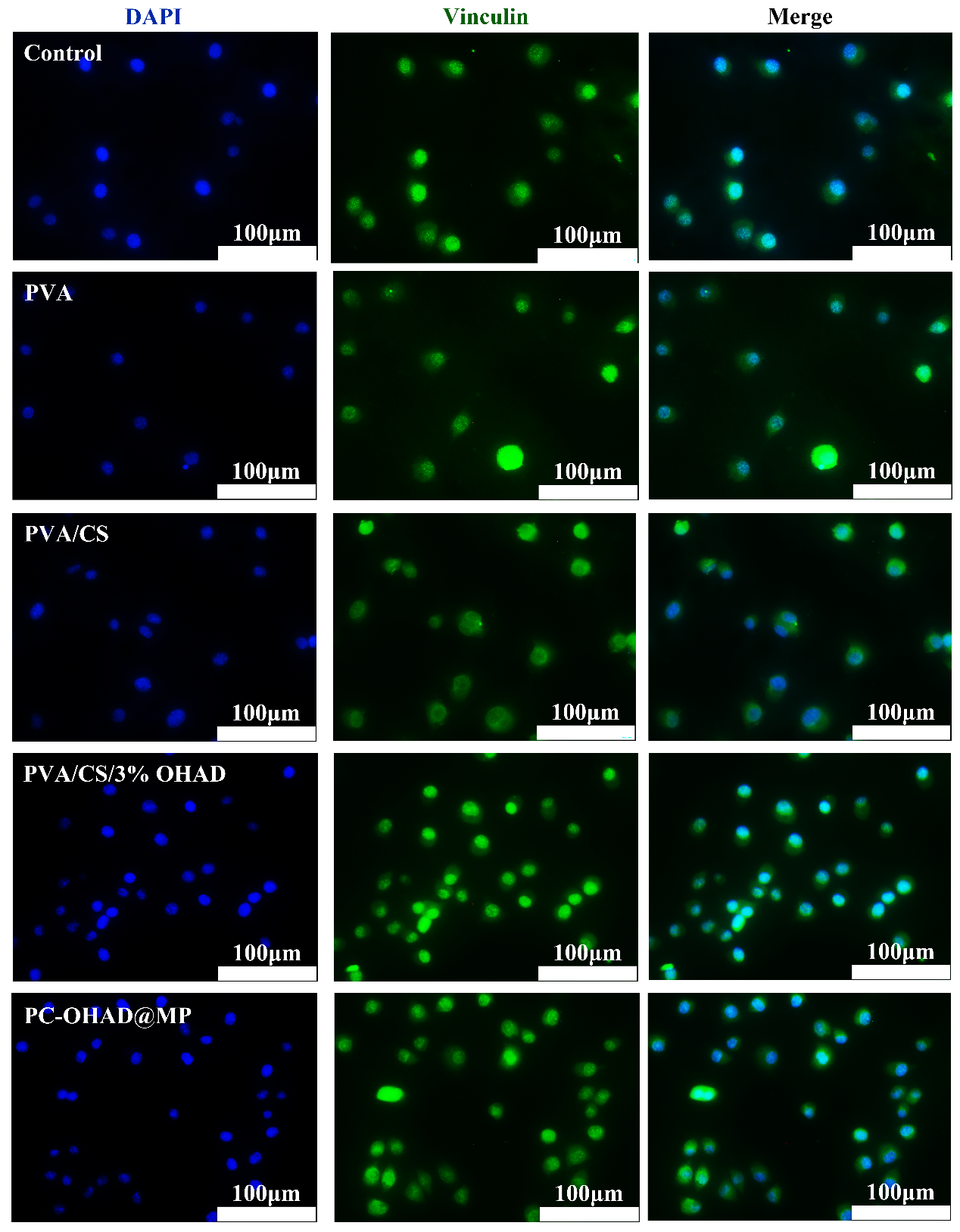


***Fig S5****. Vinculin immunofluorescence results (n = 3).*
